# Post-traumatic osteomyelitis in Middle East war-wounded civilians: resistance to first-line antibiotics in selected bacteria over the decade 2006–2016

**DOI:** 10.1186/s12879-019-3741-9

**Published:** 2019-01-31

**Authors:** Fabien Fily, Jean-Baptiste Ronat, Nada Malou, Rupa Kanapathipillai, Caroline Seguin, Nagham Hussein, Rasheed M. Fakhri, Céline Langendorf

**Affiliations:** 1 Epicentre, 55 rue Crozatier, 75012 Paris, France; 2 Infectious Diseases Unit, Broussais Hospital, Saint Malo, France; 3 Médecins Sans Frontières, 8 rue St Sabin, 75011 Paris, France; 4 Médecins Sans Frontières, New York, USA; 5 Médecins Sans Frontières, Amman, Jordan

**Keywords:** Osteomyelitis, Bone biopsy, War-wounded, First-line antibiotics resistance, Middle East

## Abstract

**Background:**

War-wounded civilians in Middle East countries are at risk of post-traumatic osteomyelitis (PTO). We aimed to describe and compare the bacterial etiology and proportion of first-line antibiotics resistant bacteria (FLAR) among PTO cases in civilians from Syria, Iraq and Yemen admitted to the reconstructive surgical program of *Médecins Sans Frontières* (MSF) in Amman, Jordan, and to identify risk factors for developing PTO with FLAR bacteria.

**Methods:**

We retrospectively analyzed the laboratory database of the MSF program. Inclusion criteria were: patients from Iraq, Yemen or Syria, admitted to the Amman MSF program between October 2006 and December 2016, with at least one bone biopsy sample culture result. Only bone samples taken during first orthopedic surgery were included in the analysis. To assess factors associated with FLAR infection, logistic regression was used to estimate odds ratio (ORs) and 95% confidence intervals (CI).

**Results:**

558 (76.7%) among 727 patients included had ≥1 positive culture results. 318 were from Iraq, 140 from Syria and 100 from Yemen. Median time since injury was 19 months [IQR 8–40]. Among the 732 different bacterial isolates, we identified 228 *Enterobacteriaceae* (31.5%), 193 *Staphylococcus aureus* (26.3%), 99 *Pseudomonas aeruginosa* (13.5%), and 21 *Acinetobacter baumanii* (2.8%). Three hundred and sixty four isolates were FLAR: 86.2% of *Enterobacteriaceae,* 53.4% of *Pseudomonas aeruginosa*, 60.5% of *S. aureus* and 45% of *Acinetobacter baumannii*. There was no difference in bacterial etiology or proportion of FLAR according to the country of origin. In multivariate analysis, a FLAR infection was associated with an infection of the lower extremity, with a time since the injury ≤12 months compared with time > 30 months and with more than 3 previous surgeries.

**Conclusions:**

*Enterobacteriaceae* were frequently involved in PTO in war wounded civilians from Iraq, Yemen and Syria between 2006 and 2016. Proportion of FLAR was high, particularly among *Enterobacteriaceae*, regardless of country of origin.

**Electronic supplementary material:**

The online version of this article (10.1186/s12879-019-3741-9) contains supplementary material, which is available to authorized users.

## Background

Post-traumatic and diabetes mellitus-related infections are the most frequent causes of osteomyelitis [1, 2]. Two retrospective studies found a rate of post-traumatic osteomyelitis (PTO) after limb fracture of 2.5 and 1.5%, but incidence of PTO depends on the host condition and on the severity of injury; it is much higher after open fractures [3, 4]. The gold standard for diagnosis of PTO is culture of bone biopsy, as deep wound tissue culture lacks sensitivity and specificity [5]. The most frequent causative pathogen is *Staphylococcus aureus*, involved in 36.2 to 57.0% of cases [2, 4–6]. Management of PTO is complex and combines initial aggressive debridement, prolonged antibiotic therapy and often reconstruction of bone or soft tissue defects [7].

Since 2003, Middle Eastern countries have experienced many conflicts, leaving thousands of civilians injured particularly in Iraq and more recently in Syria and Yemen. The majority of injuries involves extremities and is caused by bomb blasts and ballistic trauma. Due to the damage to connective tissues with open fractures, contamination with environmental debris and difficult access to timely optimal surgical care, PTO is a frequent complication of injuries in the context of conflict. This has been previously documented in the reconstructive surgical program set up by *Médecins Sans Frontières* (MSF) in Amman. Among 1353 war-wounded civilians with bone injury admitted to the program (where bone culture are taken routinely on initial surgery), 63.5% had positive bone cultures. This included patients without any clinical or radiological signs of osteomyelitis (76/167 patients, 46%) [8]. PTO, particularly in this context, may be due to commensal flora or environmental contamination at the time of injury, but may also be due to bacteria transmitted during hospitalization [9]. The spread of multi-drug resistant bacteria (MDR), usually described as organism non-susceptible to at least one agent in 3 or more antimicrobial classes [10], is a concern worldwide [11]. In the Middle East region, high rates of fecal colonization with Extended-Spectrum Beta-lactamase-Producing *Enterobacteriaceae* (ESBL-PE) has been reported in the community [12, 13], and particularly in war-wounded and refugees: 69% of 24 Syrian war-wounded children at first admission [14] and 35.1% of 134 adult refugees in four Swiss centers [15]. A high proportion of MDR has also been reported in community-acquired infections, with up to 49.4% of *Escherichia coli* isolated from urine specimens taken from outpatients and hospitalized patients in a recent meta-analysis in Iran [16, 17].

Our primary objective was to describe and compare the bacterial etiology and proportion of first-line antibiotics resistant bacteria among PTO in war-wounded civilians from Syria, Iraq and Yemen admitted to the reconstructive surgical program of MSF in Amman, Jordan, in the period October 2006–December 2016. Our second objective was to identify risk factors for an infection with FLAR bacteria.

## Methods

### Study population and microbiological analysis

In October 2006, MSF set up a surgical program in Amman, Jordan, to respond to the needs of war-wounded Iraqi civilians. As conflict expanded to neighboring countries, the program admitted patients from Yemen (since 2010) and Syria (since 2011). Patients had undergone one or multiple previous surgeries in their countries of origin, and entered the MSF program for different conditions including non-union, mal-union, chronic osteomyelitis or amputation stumps needing revision. They were referred for surgical management, and to access physiotherapy and psychological counseling. During orthopedic surgery, the surgeon collected 3 to 5 samples of bone or deep soft tissue and placed them in separate sterile containers. Antibiotic treatment was supposed to stop at least 2 weeks before the surgery. Up to August 2015, each sample was labeled and stored at 4–8 °C before being sent, within 3 h of collection, to laboratories outside the program. After this date, the cultures were carried out in the laboratory set up by MSF within the program. Bacterial culturing and identification followed the European Society of Clinical Microbiology and Infectious Diseases recommendations [18]. Antibiotic susceptibility was determined using Kirby Bauer disk diffusion, as recommended by the European Committee on Antimicrobial Susceptibility Testing [19].

### Study design and eligibility criteria

We conducted a retrospective analysis of the laboratory database entered in WHONET, software developed as shareware by the World Health Organization [20]. The inclusion criteria were: civilian patients with bone injury admitted in the Amman MSF program for orthopedic surgery between October 2006 and December 2016, of Iraqi, Syrian or Yemeni nationality, with bone tissue samples collected at time of surgery. Only bone samples taken during first surgery in the Amman MSF program were included in the analysis. We excluded soft tissue samples. Cases of PTO, defined as war-wounded patients with at least one positive bone tissue culture, were described and compared according to country of origin. If the same organism was isolated many times during the same episode of osteomyelitis, it was taken into account only once in the analysis.

### Data and statistical analysis

Based on the therapeutic guidelines currently used in the hospital, we defined a first-line antibiotics resistant bacteria (FLAR) as any of the following: i) *Enterobacteriaceae* resistant to third-generation cephalosporins (3GC, ceftriaxone or cefotaxime or ceftazidime), ii) *Pseudomonas aeruginosa* resistant to ceftazidime, iii) *Acinetobacter baumannii* resistant to carbapenems (imipenem or meropenem), or iv) *Staphylococcus aureus* resistant to oxacillin or to cefoxitin (methicillin-resistant *S. aureus*, MRSA).

Socio-demographic data of participants were described using percentages for qualitative variables and medians with Inter-Quartile Ranges [IQRs] for quantitative variables. We compared the bacterial etiology and drug resistance proportions according to country of origin using the Chi-square test or Fisher’s exact test. Means of the 3 groups were compared using the ANOVA test.

We described the evolution of the proportion of FLAR (among *Enterobacteriaceae* and *S. aureus*) over the time from January 2007 to December 2016 and cases were grouped into five periods of 2 years.

To assess factors associated with infection by FLAR bacteria, logistic regression was used to estimate odds ratio (ORs) and 95% confidence intervals (CI). Variables associated with FLAR infection with *p*-value < 0.2 in bivariate analysis were selected for inclusion in the multivariate model. Then, a back-step selection procedure was used to keep variables with a *p*-value < 0.05. Age and sex were left in the final model. Missing values were imputed with the median for quantitative variables and the mode for categorical variables.

Patients with missing values for main explicative variables were compared with other patients for the main demographic and clinical characteristics.

Analyses were done using R software.

### Ethics approval and consent to participate

This research fulfilled the criteria set by the *Médecins Sans Frontières* Ethics Review Board (MSF ERB) for review exemption as a retrospective analysis of routinely collected and anonymized data. The need for MSF ERB submission was deemed unnecessary according to MSF ERB policy.

## Results

A total of 727 patients were included, among whom 558 (76.7%) had one or more positive bone tissue cultures. Median age was 31 years and median time between injury and positive bone sample culture was 19 months [IQR 8–40]. Iraqi patients were older, with a longer delay since injury, with a greater number of previous surgeries before entering the program and with a more frequent polymicrobial infection (Table 1).

**Table 1:** Demographic and clinical characteristics of patients with war-related post-traumatic osteomyelitis according to country, 2006–2016

	Iraq (*N* = 318)	Syria (*N* = 140)	Yemen (*N* = 100)	*p*	Total (*N* = 558)	
	*n* (%)	*n* (%)	*n* (%)		*N*	*n* (%)
Male	276 (86.8)	133 (95.0)	91 (91.0)	.02	557	500 (89.6)
Age (years)^a^	34 [27–43]	26 [21.6–36]	29 [21.5–32.5]	.001	557	31 [23–40]
Diabetes	16 (5.1)	4 (3.0)	0 (0.0)	.057	527	20 (3.8)
Location of infection				.08	455	
Upper extremity	31 (14.3)	32 (23.0)	21 (21.0)			84 (18.4)
Lower extremity	185 (85.6)	107 (76.9)	79 (79.0)			371 (81.5)
Previous surgeries^a^	4 [3–7]	3 [1–5]	3 [2–5]	.017	426	4 [2–6]
Delay since injury^b^	30 [12–64]	8 [4–16]	20 [11–36]	.001	466	19 [8–40]
Fixation				.89	423	
Internal	68 (32.9)	49 (37.4)	29 (34.1)			146 (34.5)
External	71 (34.2)	42 (32.0)	31 (36.4)			144 (34.0)
No fixation	68 (32.9)	40 (30.5)	25 (29.4)			133 (31.4)
Polymicrobial infection	69 (21.7)	23 (16.4)	16 (16.0)	.02	558	108 (19.3)

^a^Quantitative variables are expressed with median [IQR]

^b^time between injury and bone tissue sample (months)

**Fig. 1:**
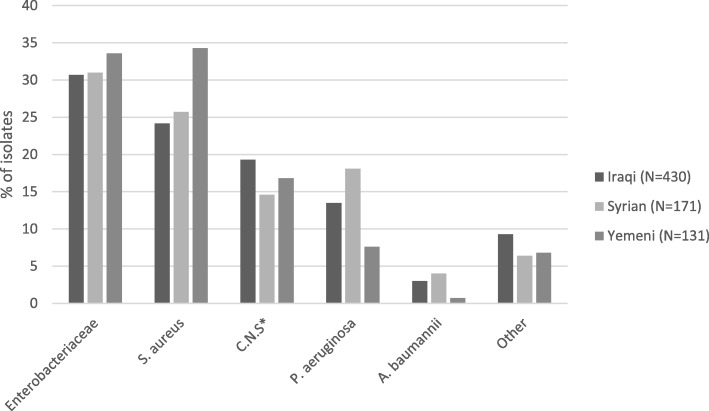
Percentage of micro-organism among total isolates from bone biopsies in war-wounded civilians according to country of origin, 2006–2016. *coagulase negative Staphylococcus

Among the 558 cases of PTO, 450 were monomicrobial and 108 were polymicrobial. A total of 732 different bacterial isolates were identified. *Staphylococcus aureus* was the most frequently isolated bacteria (*n* = 193, 26.3%), followed by *coagulase-negative Staphylococcus* (*n* = 130, 17.7%), *Escherichia coli* (*n* = 126, 17.2%), *Pseudomonas aeruginosa* (*n* = 99, 13.5%), *Klebsiella pneumoniae* (*n* = 50, 6.8%) and *Acinetobacter baumanii* (*n* = 21, 2.8%). *Enterobacteriaceae* (*Escherichia coli*, *Enterobacter cloacae, Klebsiella* spp.*, Morganella morganii* and *Proteus* spp.) represented 31.5% of all isolates (*n* = 229) and were cultured in 200 (35.8%) cases of PTO. The proportion of *S. aureus* was higher in patients from Yemen (34.3%) compared to patients from Syria (25.7%) and from Iraq (24.2%), but this difference was not statistically significant (*p* = 0.08) (Fig. 1). Bone sample cultures of the lower extremities were more frequently positive for *Enterobacteriaceae* (38.5%) compared to upper extremities (20.2%, *p* = 0.003).

According to our definition, 364 isolates were considered as FLAR. Overall, the proportion of resistance to 3GC among *Enterobacteriaceae* was 86.2% (89.7% among *E. coli* and 87.7% among *K. pneumoniae*). Nine isolates (5 *E. coli*, 2 *K. pneumoniae*, 1 *E. cloacae* and 1 *P. mirabilis*) were also resistant to imipenems (4.3% of all *Enterobacteriaceae* with susceptibility test results available). The proportion of MRSA among *S. aureus* was 60.5%. The proportion of *P. aeruginosa* isolates resistant to ceftazidime was 53.4 and 45% of *A. baumannii* isolates were carbapenem-resistant. The proportion of MRSA was not significantly different between Iraq (66.6%), Syria (52.2%) and Yemen (54.5%, *p* = 0.17). There was also no difference in the proportion of FLAR among *Enterobacteriaceae* and *P. aeruginosa* by country (Table 2).

**Table 2:** Proportion of first-line antibiotics resistant bacteria in war-related post-traumatic osteomyelitis according to country of origin, 2006–2016

Organism	Iraq		Syria		Yemen		*p*	Total	
	*N* ^a^	*n* (%)	*N* ^a^	*n* (%)	*N* ^a^	*n* (%)		*N* ^a^	*n* (%)
3GC-RE^b^	129	111 (86.0)	52	45 (86.5)	44	38 (86.3)	.9	225	194 (86.2)
MRSA^c^	102	68 (66.6)	44	23 (52.2)	44	24 (54.5)	.17	190	115 (60.5)
CRPA^d^	48	27 (56.2)	28	14 (50.0)	10	5 (50.0)	.8	86	46 (53.4)
CRAB^e^	13	4 (30.7)	6	4 (66.6)	1	1 (100.0)	.2	20	9 (45.0)

^a^Number of isolates with susceptibility test result available for each type of bacteria

^b^*Enterobacteriaceae* resistant to third-generation cephalosporin

^c^*S. aureus* resistant to methicillin

^d^*P. aeruginosa* resistant to ceftazidime

^e^*A. baumannii* resistant to carbapenem

**Fig. 2:**
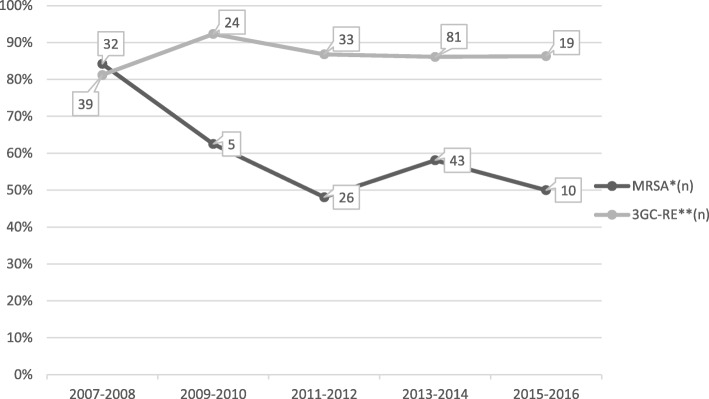
Trends of methicillin-resistant *Staphylococcus aureus* and third-generation cephalosporin resistant *Enterobacteriaceae* from war-related osteomyelitis in civilians from Iraq, Syria and Yemen. *methicillin-resistant Staphylococcus aureus, ** Enterobacteriaceae resistant to 3rd-generation cephalosporin

While the proportion of 3GC-resistant *Enterobacteriaceae* remained consistently above 80% since 2007, there was a decreasing trend in the proportion of MRSA over the period, from more than 80% in 2007–2008 to less than 60% since 2011 (Fig. 2). Eight among the 9 *Enterobacteriaceae* resistant to carbapenems were isolated after 2011.

Concerning resistance to other antibiotics of interest, 58.5% of *Enterobacteriaceae* and 50.0% of *P. aeruginosa* were resistant to ciprofloxacin, 44.3 and 41.8% of *S. aureus* were resistant to rifampin and to ciprofloxacin respectively (Table 3). These proportions were significantly (*p* < 0.01) higher among FLAR than non-FLAR isolates: 63.3% of 3GC-resistant *Enterobacteriaceae* were also resistant to ciprofloxacin (versus 24.0% in non-FLAR *Enterobacteriaceae*) as well as 66% of ceftazidime-resistant *P. aeruginosa* (vs 34.2% in non-FLAR *P. aeruginosa*); 66.6% of MRSA were resistant to rifampin (vs 17.1% in non-MRSA) and 59.0% were resistant to ciprofloxacin (vs 16.2%).

**Table 3:** Drug resistance in isolates from bone biopsy cultures in war-wounded civilians from Iraq, Syria and Yemen

	*Enterobacteriaceae*		*S. aureus*		*P. aeruginosa*	
	*N* ^a^	*n* (%)	*N* ^a^	*n* (%)	*N* ^a^	*n* (%)
Pip/tazo^b^	200	90 (45.0)	–	–	84	21 (25.0)
Cefepime	200	176 (88.0)	–	–	77	67 (87.0)
Imipenem	208	9 (4.3)	–	–	95	6 (6.3)
Ertapenem	85	8 (9.4)	–	–	–	–
Ciprofloxacin	200	117 (58.5)	177	74 (41.8)	90	45 (50.0)
Cotrimoxazole	179	150 (83.8)	144	51 (35.4)	–	–
Erythromycin	–	–	136	74 (54.4)	–	–
Clindamycin	–	–	169	86 (50.8)	–	–
Rifampin	–	–	149	66 (44.3)	–	–
Fusidic acid	–	–	142	69 (48.6)	–	–

^a^Number of isolates with susceptibility test result available

^b^Piperacillin/tazobactam

FLAR infection was diagnosed in 303 (55.2%) cases. In multivariate analysis, after adjustment for age and sex, FLAR infection was associated with an infection with the lower extremity (*p* = 0.001), with a time since the injury ≤12 months compared with > 30 months (*p* = 0.004), and with more than three previous surgeries (*p* = 0.002) [Additional file 1]. One hundred and thirty two (23.6%) patients had missing values for the number of previous surgeries, 103 (18.4%) had missing values for the site of infection and 92 (16.4%) had also missing values for the time since injury. Compared to other patients, patients with missing values were not different in terms of sex (86% male among patients with missing values vs 90.1%, *p* = 0.12), their mean age (31.2 years vs 33 years, *p* = 0.15) and the percentage with FLAR infection (53.6% vs 55%, *p* = 0.9). However, patients with missing data were more frequently from Iraq (81.6% vs 49%, *p* = 0.005).

## Discussion

PTO in war-wounded patients is a frequent and difficult to treat infection that can have serious functional consequences and even be life-threatening. We report on the bacterial etiology of PTO and the antibiotic resistance of isolates from civilians at the time they were referred from Iraq, Yemen and Syria by analyzing results of bone biopsy cultures. As expected, *S. aureus* was the most frequent pathogen isolated. However, we also found a high proportion of Gram-negative organisms and a predominance of *Enterobacteriaceae*. Our results, based here on gold standard specimen for diagnosis of PTO, is similar to that already reported in case series of osteomyelitis in war-wounded patients [21–23]. In other contexts, the proportion of *Enterobacteriaceae* in PTO is variable. This group of bacteria was involved in 2.8% of 142 PTO in one American trauma center [6], but in 35.5% of cases in another recent study in China where patients, like those in the Amman MSF program, were frequently suffering from open fracture and/or soft tissue damage following direct trauma [4]. *P. aeruginosa* was frequent in our study (13.5% of isolates); this is worrisome for PTO patients in Amman given the fact that infections with *P. aeruginosa* have been found to be associated with increased risk of recurrence of osteomyelitis [3, 6].

Over the 10 year period, half of the isolates were FLAR. Resistance to 3GC among *Enterobacteriaceae* was particularly high. This resistance is commonly associated with production of Extended-Spectrum Beta-lactamase and less frequently with AmpC cephalosporinases [24]; however we cannot specify the mechanism due to the absence of reliable information in our database. These results are in line with previous reports of infections in war-wounded in the Eastern Mediterranean and Middle East regions: 100% of 9 *Enterobacteriaceae* isolates from wounds in the combatants in the Libyan uprising in 2011 were resistant to 3GC [25]; 20 (69%) of 29 g-negative bacteria isolated in 2015 from wound (mostly with deep wound swabs) in refugees from Syria in Jordan were MDR [26]. In a study reporting results of blood culture in patients referred in a regional referral burn care center supported by MSF in Iraq in 2009, 8 (61.5%) of 13 *Enterobacteriaceae* were EBLS-PE and 12 (54.5%) of 22 *P. aeruginosa* were resistant to ceftazidime [27]. Results are similar in children: among 24 Syrian war-wounded children, admitted mostly for osteomyelitis and surgical site infection, 66% were infected with ESBL-PE [28]. Carbapenem resistance was quite rare in our series, but emerged from 2012 and is expected to increase given the global dissemination of carbapenemase-producing *Enterobacteriaceae* [29]. In our study, 60.5% of *S. aureus* isolated were resistant to oxacillin. Although we observed a slight decline over the study period, this proportion is higher than that reported in recent series of PTO in Brazil (35.5%) and in China (35.9%) [3, 4]. Resistance to other drugs of interest frequently used for osteomyelitis was also common in our patients. The percentage of resistance to fluoroquinolone, the cornerstone of treatment of bone and joint infections involving gram-negative pathogens, and particularly of prosthetic joint infections [30], was higher than 50% in *Enterobacteriaceae* and *P. aeruginosa.* The percentage of resistance to rifampin and to fluoroquinolone was higher than 40% in *S. aureus* isolates. These drugs are commonly used is osteomyelitis and are recommended as first-line oral treatment for *Staphylococcus spp.* prosthetic joint infections [31]. Due to the high rates of resistance, treatment of PTO is complex in the MSF program in Amman and requires strong antibiotic stewardship procedures as well as second-line antibiotics, often in prolonged intravenous regimens.

The origin of FLAR bacteria isolated from bone sample cultures in these patients in the MSF program is unclear. Intra-hospital transmission of MDR is common and may occur from patient-to-patient, via healthcare workers, or from the environment [32], particularly in the context of conflict with resulting instability and disorganization of health care structures [9]. But it may also be related to colonization prior to injury; the frequent carriage of MDR in the healthy population [12–15] and the high prevalence of MDR in different community-acquired infections in the Middle East region [16, 17] support this hypothesis. Our analysis found a higher number of previous surgeries to be an independent risk factor for FLAR infection. This finding has already been reported in the previous study from the MSF program in Amman [21]. However, we also found a shorter time since injury to be an independent risk factor, which seems paradoxical with the hypothesis of nosocomial transmission as the origin of FLAR. This may potentially be due to reverse causality; if it is hypothesized that pre-injury colonization with FLAR is the origin of subsequent FLAR bone infection, the initial infection would likely to be inefficiently controlled with first-line antibiotic therapy and would therefore require a higher number of surgical interventions and an earlier referral to our program. Alternatively, a survival bias could exist: patients with wound-related infections caused by FLAR may have poorer early outcome with earlier and more frequent fatal sepsis complications or requirement for amputation compared with those with infection caused by non-FLAR.

The main limitation of our study is related to the laboratory origin of the database and therefore the lack of clinical or radiological data, yet useful for the diagnosis of osteomyelitis, especially when potential contaminant like *coagulase-negative Staphylococcus* are isolated. However we limited this by analyzing only cultures from bone specimens, and not including deep soft tissue samples. Our risk factor analysis should be interpreted with caution given the number of missing values, but the association between number of previous surgeries and infection with MDR has been already reported [23]. Patients with missing data were more frequently from Iraq and we found that the time since injury was significantly longer in patients from Iraq. This could result in selection bias and an overestimation of the association between FLAR infection and shorter time since injury.

## Conclusions

Over 10 years, *Enterobacteriaceae* were frequently involved in more than 500 PTO in war-wounded civilians from Iraq, Syria and Yemen admitted to the reconstructive surgical program of MSF in Amman. Resistance to first-line antibiotics was frequent among *S. aureus* and *Enterobacteriaceae,* regardless of country of origin*.* Treatment of PTO in the Middle East war context is a challenge given the limited choice of effective antibiotics, the frequent large soft tissue damage and, more globally, the disorganization of health care systems. A high-quality laboratory, a skilled surgical team, a robust antibiotic stewardship and effective infection prevention and control practices are the cornerstones for adequate management.

## Additional file

Additional file 1: Factors associated with first-line antibiotics resistant bacteria infection in war-related post-traumatic osteomyelitis from Iraq, Syria and Yemen, 2006–2016. (DOCX 33 kb)

## Abbreviations

3GC: Third-generation cephalosporin
CI: Confidence interval
ESBL-PE: Extended-Spectrum Beta-lactamase-Producing *Enterobacteriaceae*
FLAR: First-line antibiotics resistant bacteria
IQR: Interquartile range
MDR: Multi-drug resistant bacteria
MRSA: Methicillin-resistant *Staphylococcus aureus*
MSF: *Médecins Sans Frontières*
OR: Odd ratio
PTO: Post-traumatic osteomyelitis
