# Supplementary material for: Post-traumatic osteomyelitis in Middle East war-wounded civilians: resistance to first-line antibiotics in selected bacteria over the decade 2006–2016
Source: BMC Infect Dis. 2019 Jan 31;19:103. doi: 10.1186/s12879-019-3741-9 (PMC6357381; doi:10.1186/s12879-019-3741-9)
Supplement: Supplementary file 1 — Factors associated with first-line antibiotics resistant bacteria infection in war-related post-traumatic osteomyelitis from Iraq, Syria and Yemen, 2006–2016. (DOCX 33 kb) [file 12879_2019_3741_MOESM1_ESM.docx]

Table S1: Factors associated with first-line antibiotics resistant bacteria infection in war-related post-traumatic osteomyelitis from Iraq, Syria and Yemen, 2006-2016.

|  | **FLAR**^a^ | **Bivariate analysis** | | | **Multivariate analysis** | | |
| --- | --- | --- | --- | --- | --- | --- | --- |
|  | n (%) | OR | 95%CI | p | OR | 95%CI | p |
| **Sex** |  |  |  |  |  |  |  |
| Female | 30 (54.5) | 1 |  | .9 | 1 |  | .69 |
| Male | 273 (55.3) | 1.03 | 0.58-1.80 |  | 1.12 | 0.62-2.01 |  |
| **Age** |  |  |  |  |  |  |  |
| **>**30 years | 154 (53.6) | 1 |  | .42 | 1 |  | .49 |
| ≤30 years | 149 (57.0) | 1.15 | 0.82-1.61 |  | 0.88 | 0.62-1.26 |  |
| **Diabetes** |  |  |  |  |  |  |  |
| No | 277 (55.6) | 1 |  | .41 | - | - | - |
| Yes | 13 (65.0) | 1.48 | 0.59-4.00 |  | - | - | - |
| **Location of surgery** |  |  |  |  |  |  |  |
| Upper extremity | 33 (39.2) | 1 |  | .002 | 1 |  | .001 |
| Lower extremity | 270 (58.0) | 2.14 | 1.33-3.47 |  | 2.24 | 1.36-3.73 |  |
| **Delay**^b^ |  |  |  |  |  |  |  |
| >30 months | 78 (50.6) | 1 |  | .07 | 1 |  | .004 |
| 12-30 months | 119 (52.2) | 1.09 | 0.72-1.64 |  | 1.04 | 0.67-1.60 |  |
| ≤12 months | 106 (61.9) | 1.61 | 1.03-2.51 |  | 1.97 | 1.23-3.20 |  |
| **Previous surgeries** |  |  |  |  |  |  |  |
| ≤3 | 93 (46.5) | 1 |  | .008 | 1 |  | .002 |
| 4-5 | 64 (58.2) | 1.67 | 1.04-2.69 |  | 1.88 | 1.15-3.10 |  |
| >5 | 146 (60.5) | 1.76 | 1.21-2.58 |  | 1.94 | 1.30-2.92 |  |
| **Fixation** |  |  |  |  |  |  |  |
| Internal | 74 (51.3) | 1 |  | .47 | - | - | - |
| External | 81 (56.6) | 1.23 | 0.77-1.97 |  | - | - | - |
| Non | 77 (58.3) | 1.32 | 0.82-2.13 |  | - | - | - |
| **Country of origin** |  |  |  |  |  |  |  |
| Yemen | 52 (52.0) | 1 |  | .67 | - | - | - |
| Syria | 74 (54.0) | 1.08 | 0.64-1.81 |  | - | - | - |
| Iraq | 177 (56.7) | 1.21 | 0.77-1.90 |  | - | - | - |
| ^a^ First-line antibiotics resistant bacteria ^b^ time between injurie and bone sample culture | | | | | | | |
